# Evaluating the impact of three progestin-based hormonal contraceptive methods on immunologic changes in the female genital tract and systemically (CHIME Study): a prospective cohort study protocol

**DOI:** 10.1186/s12905-022-02053-w

**Published:** 2022-11-18

**Authors:** Lisa B. Haddad, Gina Bailey Herring, C. Christina Mehta, Tyree Staple, Marisa R. Young, Sakthivel Govindaraj, Vijayakumar Velu, Alicia K. Smith

**Affiliations:** 1 Center for Biomedical Research, Population Council, New York, NY USA; 2 Department of Gynecology and Obstetrics, School of Medicine, Emory University, 101 Woodruff Circle NE, GA 30322 Atlanta, USA; 3 Department of Epidemiology, Rollins School of Public Health, Emory University, Atlanta, GA USA; 4 Grady Infectious Disease Program, Grady Health System, Atlanta, GA USA; 5 Division of Infectious Diseases, Department of Medicine, School of Medicine, Emory University, 101 Woodruff Circle NE, Atlanta, GA 30322 USA; 6 Department of Pathology and Laboratory Medicine, School of Medicine, Emory University, Atlanta, GA USA; 7 Division of Microbiology and Immunology, Emory Vaccine Center, Emory National Primate Center, Emory University, Atlanta, GA USA

**Keywords:** Progestin contraception, Immunology, Female genital tract, Vaginal microenvironment, HIV target cells

## Abstract

**Background:**

Gonadal hormones can modify immune function, which may impact susceptibility to infectious diseases, including Human Immunodeficiency Virus (HIV). There is limited knowledge about how hormonal contraceptives (HC) influence the immune response during the course of use. The CHIME study aims to evaluate the effect of long-acting progestin-based hormonal contraceptives (depot medroxyprogesterone acetate, etonogestrel implant, and levonorgestrel intrauterine device) on immunologic changes in the female genital tract (FGT) and systemic compartment.

**Methods:**

CHIME is an observational cohort study where participants attend 2 visits prior to initiating the HC method of their choice, and then attend 6 visits over 12 months with biological sampling (vaginal swabs, cervicovaginal lavage, cytobrush and blood) for immunological, bacteriological, and virological analyses at each visit. Immune profiling will be evaluated by multi-color flow cytometry to determine how different T-cell subsets, in particular the CD4 T-cell subsets, change during the course of contraceptive use and whether they have different profiles in the FGT compared to the systemic compartment. The study aims are (1) to characterize the alterations in FGT and systemic immune profiles associated with three long-acting progestin-only HC and (2) to evaluate the vaginal microenvironment, determined by 16 s rRNA sequencing, as an individual-level risk factor and moderator of genital and systemic immune profile changes following exposure to three commonly used HC. Data collection started in March 2019 and is scheduled to be completed in October 2024.

**Discussion:**

The CHIME study aims to contribute to the body of research designed to evaluate the comparative impact of three long-acting progestin-only HC on innate and adaptive immune functions to understand how immunologic effects alter STI and HIV susceptibility.

## Background

Approximately 40% of all pregnancies worldwide are unintended, with an estimated 50% ending in abortion [1]. Contraception reduces infant mortality, empowers women, enhances educational attainment, reduces the burden of unintended pregnancy, and dampens the impact of population growth [2]. The most common forms of contraception used worldwide are progestin-containing hormonal contraception (HC), either alone or in combination with estrogen, with over 150 million women using HC methods to achieve family planning goals [3, 4].

There have been concerns that HC, specifically depot medroxyprogesterone acetate (DMPA), may contribute to the spread of Human Immunodeficiency Virus (HIV) by increasing a woman’s susceptibility to infection [5]. One randomized control trial, the Evidence for Contraceptive Options and HIV Outcomes (the ECHO trial), found no statistically-significant differences in HIV acquisition between DMPA, contraceptive implant, and the copper intrauterine device (IUD) [6]. While the ECHO trial has provided valuable evidence, the trial was not able to address the risk of other contraceptive methods not included nor evaluate the mechanism of the purported HIV risk. Additional mechanistic studies are needed, and long-acting contraceptive methods, including the levonorgestrel (LNG) IUD and etonogestrel (ENG) implant, deserve evaluation to fill the knowledge gap and allow for informed decision making.

HC methods employ different strategies to prevent pregnancy, including ovulation inhibition, altered endometrial structure, and thickening the cervical mucous barrier. Common progestin-containing contraceptives differ by the type of progestin they contain, mode of delivery, length of effectiveness, global availability, and degree of endogenous hormone and ovulation inhibition (Table 1). Each progestin has a different potency; half-life; and degree of estrogenic, androgenic, anti-androgenic, glucocorticoid, and anti-mineralocorticoid activity [7, 8]. DMPA, ENG-implant, and LNG-IUD were selected as the long-acting (three months or longer) methods for this study for several reasons. Globally, the rate of DMPA use is high, and data is most available for this method, thus serving as an imperative comparator to the other methods. The ENG-implant (the only implant available in the US) and the LNG-IUD have become increasingly popular [9]; however, there is minimal data on the impact of these methods on immunologic effects and HIV risk.

**Table 1:** Comparative properties of three-progestin-only contraceptive methods used in this study

	**Depot Medroxyprogesterone Acetate (DMPA)**	**Levonorgestrel IUD**	**Etonogestrel Implant**
**Trade-name**	DepoProvera	Liletta	Nexplanon
**Progestin**	Medroxyprogesterone acetate (MPA) 1st generation	Levonorgestrel (LNG) 2nd generation	Etonogestrel (ENG) 3rd generation
**Delivery**	Intramuscular Injection	Intrauterine Device	Sub-dermal Implant
**Length of Use**	3 months	6 years	3 years
**Global Utilization**	High	Low	Low
**Ovulation Inhibition**	High	Low	High
**Endogenous estrogen inhibition**	Yes	No	Some initially

A significant difference between these methods is the kinetics of systemic progestin release with varying degrees of hypothalamic-pituitary-ovarian (HPO) axis modulation and differential estrogen suppression [8]. DMPA plasma concentrations peak in the week after administration, followed by a plateau of serum concentrations for about three months with suppression of endogenous estradiol and progesterone levels [10]. After a peak following ENG-implant insertion, concentrations reach steady-state levels at about 4–6 months and remain sufficient to prevent ovulation for three years. Ovarian activity slowly increases with estradiol levels in the normal range after six months; thus, serum estrogen concentrations are significantly higher in ENG-implant users compared to DMPA users [11]. A few weeks after placement of LNG-IUD, steady-state plasma LNG concentration is reached, and levels are maintained for at least five years after insertion. Ovulatory function is normal, and there are no changes in endogenous estradiol and progesterone concentrations [12]. Although systemic levels of LNG are low, the impact of higher hormone concentrations localized to the female genital tract (FGT) on immune function is unknown.

HIV infections among women can occur through heterosexual transmission within the FGT. In addition to HIV co-receptor expression, CD4 + T cells are heterogeneous with regard to their HIV susceptibility, and certain T-cell phenotypes such as activated T cells (cells expressing markers such as HLADR and CD69) and cells expressing mucosal trafficking markers (such as α4β7 and CCR7) have been associated with increased risk of HIV acquisition in a macaque model of HIV infection [13–16]. This study postulates that hormonal contraceptives, either directly and/or via their impact on endogenous sex steroid levels, modulate the expression and recruitment of HIV target cells to the female genital mucosal surface [17].

There are several mechanisms by which hormonal contraceptives may alter HIV susceptibility. High levels of estrogen and progesterone during pregnancy are associated with a shift from a TH1 to a TH2 dominant immune profile, dampening the pro-inflammatory pathways, and increasing susceptibility to certain disease conditions while reducing the severity of others [18–21]. Immune changes likely accompany supra-physiologic hormone concentrations achieved with HC, while suppressing endogenous sex hormone levels. Several studies have evaluated the immune effects of HC in the FGT. Those that have evaluated DMPA [22–36] and LNG-IUD [37–42] have had conflicting findings [24, 26–28, 32, 36]. To our knowledge, no published study has evaluated the immune effects of the ENG-implant.

Lactobacillus species are typically thought to dominate the FGT microbiome. However, one third of US women [43–45] and over 50% of non-Hispanic African American women [46] have a polymicrobial vaginal flora with increased bacterial diversity and gram stain characteristics consistent with bacterial vaginosis (BV) [27, 44]. Existing data suggest that the elevated HIV risk associated with BV is due, in part, to the recruitment of HIV target cells to the vaginal mucosa and increased inflammatory mediators [47]. While increases in BV-associated bacteria and decreases in lactobacilli species have been proposed as mechanisms for increased HIV susceptibility with HC, several studies have suggested that some HC methods might be protective against BV [48–50]. Since both HC and BV may enhance HIV risk via increases in FGT HIV target cells and inflammatory mediators, it is conceivable that these two factors act synergistically to potentiate HIV acquisition risk among women with BV who are on HC.

A diagnosis of BV often indicates an abundance of diverse microbial species and communities rather than a single species in the FGT. Historically, BV was diagnosed using clinical criteria (Amsel’s criteria) or gram stain to identify altered microbiota. Genetic sequencing technology, including 16 s rRNA sequencing, provides granular detail on genus and species alterations that previously were unavailable and may be more informative. These more sensitive microbiome techniques offer an opportunity to improve understanding of the microenvironment at the species level.

Supra-physiologic levels of exogenous sex steroids are capable of modulating FGT mucosal immunity [51–54]; however, these effects are not consistently observed across all studies [55]. Inconsistencies may be attributable to the influence of additional modifying factors, especially the vaginal microenvironment [56]. This study will test the hypothesis that the vaginal microenvironment has an important modifying role in the relationship between HC and HIV risk. Further, we seek to characterize the vaginal microenvironment using gram stain criteria and, additionally, using the more sensitive 16 s rRNA techniques to identify specific bacterial species or community state types capable of modifying HIV risk. Understanding this interplay may provide opportunities for targeted interventions to alter the composition of the vaginal environment and potentially modify the STI/HIV acquisition risk with HC.

The study tests the overarching hypothesis that hormonal contraceptives induce systemic and mucosal immune changes capable of altering susceptibility or response to diseases, including HIV infection, and that these effects vary in nature and magnitude by contraceptive type and will be modified by the vaginal microenvironment. Our study objectives are [1] to characterize the alterations in female genital and systemic immune profiles associated with three long-acting progestin-only HC methods: DMPA, ENG-implant, and LNG-IUD; and [2] to evaluate the vaginal microenvironment as a moderator of genital and systemic immune profile changes following exposure to these three commonly-used hormonal contraceptives.

## Methods

### Study design and recruitment

**Fig. 1:**
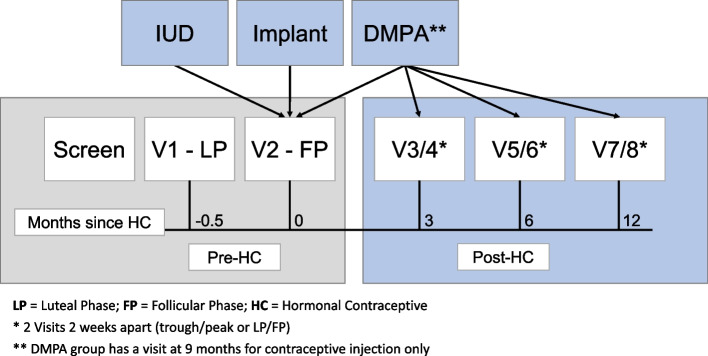
Overview of study design and time points

The CHIME study, as depicted in Fig. 1, is a prospective cohort evaluating the impact of progestin-based HC methods on immunologic changes in the FGT and systemic compartment. The study aims to recruit 225 HIV-negative women desiring HC, including 75 women in each contraceptive arm (DMPA, ENG-implant, LNG-IUD). The study has been reviewed and approved by the Emory University Institutional Review Board (IRB) and the Grady Health System Research Oversight Committee.

Subjects are recruited from several sites in the greater Atlanta community. Sites include (1) Grady Health System Family Planning clinic that provides services for healthy reproductive-aged women from across the state of Georgia; (2) referral from the Grady gynecologic and primary care clinics; (3) community-based recruitment through postings at community centers and universities; and (4) web-based recruitment through the national ResearchMatch project and Trialfacts.

### Inclusion and exclusion criteria

Individuals assigned female at birth younger than 45 years of age are eligible to participate if they (1) have normal menses (occurring within 22–35 day intervals) for at least two cycles; (2) have an intact uterus and cervix; (3) are interested in initiating HC and willing to accept DMPA, ENG-implant, or LNG-IUD; (4) willing to delay initiation of HC for up to one month; (5) able and willing to provide informed consent and undergo study procedures; (6) have a negative HIV test by OraQuick® method (OraSure Technologies, Bethlehem, PA) at the screening visit; (7) and agree to abstain from vaginal intercourse or using intra-vaginal products for one day prior to each study visit.

Women are not eligible for the study if they meet any of these criteria: (1) pregnant or planning to become pregnant within the next year; (2) breastfeeding, if not having regular, active menstrual cycles; (3) history of loop electrosurgical excision procedure (LEEP), conization, or cryosurgery within the past year; (4) current use of systemic HC or IUD, based on self-report and/or hormonal testing; (5) taking concurrent medications that interact with selected HC; (6) contraindications to selected contraceptive method using the Centers for Disease Control and Prevention Medical Eligibility Criteria or judgment of the study clinician.

### Participant timeline

Upon successful screening and confirmation of eligibility, study staff schedule participants for study visit 1 approximately three weeks following the onset of their menses (luteal phase for women who cycle every 22–35 days). The timing for visit 1 and subsequent study visits will occur according to the schedule outlined in Table 2. At each visit, an interval assessment of bleeding and hormonal contraception use is collected and used to determine the phase of the menstrual cycle where applicable. Participants choose the specific contraceptive type that is initiated at visit 2. Laboratory personnel are blinded to contraceptive choice.

**Table 2:** Participant timeline

	**Pre Contraception**			**Post Contraception**						
Study Activities	*Screen*	*Visit 1*	*Visit 2*	*Visit 3 & 4*		*Visit 5 & 6*		*Visit 6a*	*Visit 7 & 8*	
Timing of visit		*Luteal Phase*	*Follicular Phase*	*3 months*		*6 months*		*9 months*	*12 months*	
**Informed consent**	X									
**Complete history and physical exam (if indicated)**	X									
**Targeted history, questionnaire, and physical exam**		X	X	X	X	X	X		X	X
**RPR and HSV**		X								
**HIV Ora-Quick Test**	X					X			X	
**Pregnancy test**	X	X	X	X		X		X	X	
**DMPA administration**			X	X		X		X	X	
**ENG-implant or LNG-IUD placement**			X							
**Bleeding diary**			X	X						
**Genital/rectal sampling**^**a**^		X	X	X	X	X	X		X	X
**Blood sampling**^**b**^	X	X	X	X	X	X	X		X	X
**Cervical/Vaginal biopsies**^**c**^			X		X		X			X
**Flow-Cytometry**		X	X	X	X	X	X		X	X
**Cytokines & Immune-related proteins**		X	X	X	X	X	X		X	X
**HIV inhibitory activity**		X	X			X	X			

^a^Cervicovaginal lavage and cytobrush: immunologic testing, PSA; swabs: STIs, wet-mount, gram stain, microbiome, rectal microbiome; ^b^Blood: Immunologic testing, hormone panel ^c^Optional/only if subject consents

### Clinical evaluation and specimen collection

Study visits include a brief questionnaire and collection of specimens. The questionnaire covers topics including demographics, medical history, BMI, parity, smoking, vaginal practices (e.g., douching, lubricants), sex (frequency, barrier use), psychosocial and behavioral questions, and medication use, including antibiotic use. Urine is collected for a point of care pregnancy test (Consult Diagnostics, McKesson hCG test cassette, Richmond, VA) at study visits 1, 2, 3, 5, and 7. Ora-Quick® point of care HIV testing is performed at study visits 1, 5, and 7.

A trained study clinician performs the clinical evaluation and all specimen collections using standardized protocols detailed below. Oral microbiome swabs are collected at every visit; the study clinician or the participant may collect by rubbing the Puritan Dry Transport System swab along the insides of both cheeks for 20 s (Puritan Medical Systems, Guilford, ME). Additional swabs of the same type are used for collection of the vaginal and rectal microbiome samples. During the pelvic exam, vaginal pH is measured using a point of care MColorpHast™ pH test strip (EMD Millipore Corporation, Billerica, MA), a vaginal swab is collected for evaluation of vaginal microbiome, a vaginal swab is collected for a wet mount microscopic evaluation, and a dry slide is collected for gram stain and Nugent evaluation. A cervical swab for sexually transmitted infection (STI) testing (*N. gonorrhea*, *C. trachomatis*, and *T. vaginalis*) using the MultiCollect Specimen Collection swab (Abbott Molecular Inc, Des Plaines, IL) is collected at visits 1, 3, 5, and 7. Cervicovaginal fluid is collected by cervicovaginal lavage (CVL). The CVL procedure is performed during the speculum exam using 10 mL of room temperature (RT) phosphate-buffered saline (PBS) and a disposable plastic 3 mL pipette to direct the fluid toward the cervical os (the opening in the center of the ectocervix) and vaginal walls for one minute. The first 1.5 mL of the CVL is placed into a plastic microcentrifuge tube with no additive, and the remainder of the lavage is placed into a 15 mL conical tube with 5.5 mL human cell serum (GeminiBio, GBP-100–512). This procedure is performed twice to increase the potential cell yields for flow cytometry analysis. The presence of visible blood contamination is assessed in all CVL specimens at the time of sample collection. Cervical cytobrush sampling is collected by inserting the Medscand® Medical Cytobrush (CooperSurgical, Trumbull, CT) into the endocervical os, rotating the cytobrush 360 degrees, and immediately placing the cytobrush into a 50 mL conical tube with 15 mL of complete media (CM: RPMI -1640 with L-glutamine [Gibco] with 10% heat-inactivated human serum, 100 µg/mL gentamicin, 1% penicillin, and 20 mM HEPES buffer); this procedure is repeated with a second cytobrush. At visit 1, a cervical cytobroom specimen is collected for high-risk human papillomavirus (HPV) mRNA testing using the Thin-Prep® Aptima® platform (Hologic, Inc., Marlborough, MA). At visits 2, 4, 6, and 8, an optional collection of up to four cervical biopsies is performed using a Euro-Med baby Tischler gynecologic biopsy forceps (CooperSurgical, Trumbull, CT); the biopsy samples are immediately placed into a 50 mL conical tube with 15 mL of complete media. The rectal microbiome swab is collected at every study visit, and rectal STI testing is collected at visit 1; both samples are clinician-collected by inserting the appropriate swab into the rectum.

In addition to the pelvic evaluation, peripheral venous blood is collected at each visit in two 8 mL cell preparation tubes (CPT; BD Vacutainer™, Becton, Dickinson, and Company) for immunologic markers and hormonal assessment. At visit 1, additional blood is collected in a serum separator tube (SST; BD Vacutainer™, Becton, Dickinson, and Company) for herpes simplex virus (HSV) antibody serology, syphilis testing via rapid plasma reagin (RPR), and HIV antibody/antigen testing (if indicated when the Ora-Quick® is positive or indeterminant).

Collected specimens are immediately stored on ice and are processed for cellular isolation and characterization within four hours. Table 2 lists the schedule of events and biologic specimen collections for the study.

### Microbiome specimen processing

Vaginal, oral, and rectal dry transport system swabs are collected and stored at -80 °C for future microbiome characterization via next-generation sequencing of the 16S rDNA from bacterial species. Within the current study we aim to characterize the microbiome present in the vaginal compartment, however in future studies will aim to characterize those from oral and rectal swabs.

For the vaginal microbiome, microbial DNA is extracted from swabs using the DNeasy PowerSoil Kit (Qiagen), quantified with the Broad Range Quant-It kit from ThermoFisher Scientific, and assessed for quality on a 2% agarose gel. DNA is extracted with non-template controls and positive controls. Microbial composition is characterized by DNA sequencing of the V3–V4 regions of the 16S rRNA gene using the Illumina MiSeq platform and according to the manufacturer’s instructions. We anticipate a minimum of 50,000 paired end reads of high quality for each sample allowing detection of rare species and accurate quantification of common species.

QIIMEII will be used for initial sample quality control followed by processing dada2 for read assembly, taxonomy assignment of amplicon sequence variants, and classification. Total sequence counts or relative abundances are used to calculate Shannon (richness) and Chao1 measures of alpha-diversity. A community state type (CST) is assigned to each sample using hierarchical clustering with the Jensen-Shannon divergence and Ward linkage [45, 57]. CST I is predominated by L. crispatus, CST II by L. gasseri, CST III by L. iners, CST IV is defined as lacking Lactobacillus predominance and comprising a diverse set of strict and facultative anaerobes, further split into CST IV-A (predominated by BVAB and Gardnerella), IV-B (predominated by Atopobium and Gardnerella), and IV-D (only anaerobes), while CST V is predominated by L. jensenii.

### STI testing

The wet mount is prepared by the study clinician immediately following the pelvic exam using saline and 10% KOH; microscopy is used to evaluate for budding yeast, pseudohyphae, clue cells, and trichomonads. Dry slides are stored for later gram staining for Nugent scoring [58] to diagnose BV. At visits 1, 3, 5, and 7, cervical swabs are collected, and at visit 1, oropharyngeal swabs and rectal swabs are collected and sent to the Emory Clinical Virology Research Laboratory for nucleic acid amplification test (NAAT) to evaluate for *N. gonorrhea*, *C. trachomatis*, and *T. vaginalis*. Syphilis screening is determined via a commercially-available rapid plasma reagin (RPR) kit performed at the Emory Clinical Virology Research Laboratory. Serological tests for HIV and HSV are performed via enzyme immunoassay (EIA) using a Vitros® 3600 Immunodiagnostic system (Ortho Clinical Diagnostics) and ThinPrep® collections for high-risk HPV typing and genotyping are performed using the Aptima® Technology System and ThinPrep Processor (Hologic®, Marlborough, MA) at the Grady Health System CLIA-approved laboratory. Positive test results from the research lab cannot be relied upon for patient care. Any positive test results from the research labs concerning for infection triggers further clinical investigation with referral for clinical evaluation, further testing, and treatment.

### Blood processing

Peripheral blood mononuclear cells (PBMC) are isolated by centrifuging sodium-citrate-containing CPT tubes at 2500 rpm at 25 °C for 30 min. After centrifuging, the plasma is aliquoted. The PBMCs are isolated from the remaining buffy coat present in the CPT tubes. The original blood tube is vortexed, then the remaining cells are transferred in a separate 15 mL centrifuge tube, and complete media is added up to the 10 mL mark. Wash once at 1500 rpm for 5 min at RT and then aspirate for supernatant. Add 5 mL ammonium-chloride-potassium (ACK) lysing buffer (Quality Biological) and incubate for 5 min, then add complete media to a 10 mL line on the tube. Centrifuge the tube at 1500 RPM for ten minutes at RT and then aspirate for supernatant, wash twice with complete media. Resuspend the pellet in 2 mL complete media. Take 10 µl volume of cells mixed with 10 µl of trypan blue stain to check viability and cell count using Invitrogen automated cell counter (Invitrogen, Fischer Scientific, Waltham, MA). Immunophenotyping is performed using 2–3 million cells.

### CVL processing

CVL specimens are tested for prostate-specific antigen (PSA), a marker for semen exposure, using the Abacus ABAcard® p30 test (Abacus Diagnostics, West Hill, CA); testing for CVL blood and leukocyte levels is also performed with Mutistix® 8SG urinalysis strips (Siemens Healthcare, Los Angeles, CA). CVL samples are centrifuged to separate into supernatant and cellular fractions; 8 mL of the CVL supernatant is collected, and aliquots are labeled and stored at -80 °C for subsequent assays. The remaining CVL supernatant is filtered through a 100 μm cell strainer, complete media is added, and the mixture is centrifuged and aspirated for supernatant. The pellet is resuspended in complete media, and from this, 3–4 million cells are used for multi-color flow cytometry staining via standard surface staining protocol (BD LSR Fortessa). The remaining cells are mixed with freezing media (10% dimethyl sulfoxide containing 90% human serum), and aliquots are stored in a liquid nitrogen freezer (-195 °C).

### Cervical cytobrush processing

Cytobrush samples are processed according to study procedures, then stained with trypan blue stain to check viability and cell count in the automatic cell counter (Invitrogen). The complete sample is used for real time flow cytometry staining.

### Cervical biopsy specimen processing

Biopsy samples are processed into a liquified cellular sample according to study procedures. A 10 mL volume of cells is mixed with trypan blue stain to check viability and cell count in the automatic cell counter. Remaining cells are used for real time multi-color flow cytometry immunophenotyping assay.

### Immune cell characterization

Staining of PBMC, CVL, cytobrush, and cervical biopsy samples is done at RT, while the cell counts for PBMCs, cervical biopsies, cytobrush cells, and CVL cells are done at 4 °C. Cytobrush (1–2 × 10^6^) and CVL (3–4 × 10^6^) cell suspensions are stained for 30 min in PBS containing 2% FBS (fluorescence-activated cell sorting [FACS] wash buffer: 1X PBS1000 mL, 2% FBS and 500 mg sodium azide). Cells are stained with fluorochrome-conjugated antibodies from BD Pharmingen or Biolegend (San Diego, CA) specific for CD3 (SP34-2), CD4 (OKT4), CD8 (RAP-T8), CCR5 (3A9), PD-1 (EH12.2H7), Ki-67 (B56), CD38 (HIT2), CCR7 (G043H7), CD56 (NCAM16), CD45 (HI30), CD69 (FN50), Alpha4Beta7 (NHP), CD103 (2G5), HLADR (L243), and CD45RA (HI100). Dead cells are excluded from analysis based on staining for LIVE/DEAD™ Near-IR Dead Cell Stain (Molecular Probes, Invitrogen Grand Island, NY). Fox-P3 and Ki-67 stains are performed after cells are stained for surface antigens, followed by permeabilization/fixation using the Invitrogen intracellular staining protocol kit and protocol, followed by intracellular staining for 30 min at RT. Samples are washed with FACS wash buffer and centrifuged at 1500 rotations per minute (RPM) for 5 min. The samples are acquired on a LSRFortessa™ (BD Biosciences, San Jose, CA), and a minimum of 500,000 total events are collected for each sample. Using FlowJo™ software version X.0.7 (Tree Star, Inc., Ashland, OR), data is analyzed after gating out dead cells, and subsequently gating on the CD45^+^ leukocytes population. CD3^+^ cell subsets are gated, and all analyses with CD4^+^ T-cells subsets are performed on parent CD4^+^ T-cell subsets.

We characterize subsets of CD4 + T cells expressing markers of activation (HLA-DR, CD69), cell trafficking (CD103, α4β7), cell regulation (Fox-P3), proliferation (Ki67), and tissue resident (CD69, CD103). Tissue-resident markers are characterized by the expression of CD103 and CD69 [59], which bind to E-cadherin expressed in tight junctions of epithelial cells in the mucosal surface [60], a homing marker expressed on T cells at mucosal sites. CCR7 regulates migration to lymph nodes [61]. α4β7 is a heterodimeric integrin receptor involved in T cell migration into the gut-associated lymphoid tissue, which has been associated with HIV acquisition in macaque models [61]. Ki67 is a prototypical cell cycle-related protein exclusively expressed on proliferating, not quiescent cells. CD3^+^CD4^+^ T cells are analyzed for co-expression of T cell activation, trafficking, and proliferation markers and reported as a percent of total lymphocytes and total CD4^+^ cells.

### Cytokines and immune-related protein assays

A panel of soluble markers including pro-inflammatory, anti-inflammatory, inhibitory, and chemotaxic cytokines and chemokines were selected based on their ability to be measured in plasma and CVL, their role in HIV or STI risk (e.g., RANTES, MIP-1B, IL8), or their ability to influence activation or recruitment of HIV target cells to the FGT (e.g., IL1, IP10) [62–64]. Using MSD multiplex immunoassays, we evaluate CVL supernatant and plasma samples collected at each study visit.

The following cytokines and immune-related proteins are quantified using U-PLEX Biomarker Group 1 Panel (Meso Scale Diagnostics, Rockville, MD, USA) according to the manufacturer's instructions: eotaxin, GM-CSF, IFN-α2a, IFN-γ, IL-1α, IL-1β, IL-2, IL-4, IL-5, IL-6, IL-8, IL-10, IL-12/IL-23p40, IL-17A, IP-10, MCP-1, MIP-1α, MIP-1β, TNF-α, TNF-β. RANTES are quantified using the R-PLEX Human RANTES Antibody Set (Meso Scale Discovery, Rockville, MD, USA), also according to the manufacturer's instructions. All assay standards and experimental samples are run in duplicate. The plates are read on the MSD QuickPlex instrument, and the concentrations are evaluated on the MSD software platform, which converts luminescence to protein concentrations based the respective calibrator concentrations. Duplicates that vary by > 20% are repeated. Concentrations below the lower limit of detection (LLOD) for a sample is imputed as the LLOD divided by the square root of 2.

### Ex vivo* HIV inhibitory activity in FGT secretions*

While direct ex vivo HIV challenge assays can be assessed, the variability in susceptibility of FGT tissue to HIV and the need for multiple biopsies, an invasive procedure, renders this an impractical strategy. An alternative approach is to measure the antimicrobial activity in CVL [65–71] which may represent a mixture of interactions mediated by secreted cytokines, chemokines, and antibodies along with molecules secreted by the microbiota [72, 73]. To evaluate the endogenous antimicrobial activity, CVL supernatant aliquots are stored at -80 °C on the same day of collection. Samples from visits 1, 2, 5, and 6 are shipped on dry ice to the lab of Dr. Herold at Albert Einstein School of Medicine for evaluation of FGT secretion inhibitory activity. To assess inhibitory activity against HIV, TZM-bl cells are infected with HIV-1BaL (~ 103 TCID50) mixed 1:1 with CVL or control buffer (normal saline containing 200 mg/mL bovine serum albumin) and infection monitored by assaying for luciferase activity 48 h after infection.

### Hormonal testing

Plasma samples are shipped to the Endocrine Technologies Core (ETC) at the Oregon National Primate Research Center (ONPRC) for sex steroid assays. Endogenous and exogenous hormones (estradiol, estrone, estriol, ethinyl estradiol, LNG, ENG, progesterone, MPA, norethindrone, testosterone, dehydroepiandrosterone, dihydrotestosterone, androstenedione, and dihydroprogesterone) are measured simultaneously by mass spectrometry using the method developed by Blue and colleagues [74]. Luteinizing hormone (LH) and follicle-stimulating hormone (FSH) concentrations are measured using a Roche Cobas e411 automatic immunoassay (Roche Diagnostics, Indianapolis, IN). These data enable us to confirm the phase of the cycle and other exogenous hormone exposure. At screening, participant self-report regarding prior HC use is considered acceptable for eligibility purposes. Plasma hormone levels are measured, as the results could affect eligibility of data inclusion.

### Specimen and data management

All study personnel have appropriate institution-specific laboratory and human subject safety trainings. Blood and secretion precautions are employed during phlebotomy, specimen collection, and specimen handling, as currently recommended by the Centers for Disease Control and Prevention. All infectious specimens are transported using packaging mandated in the Code of Federal Regulations, 42 CFR Part 72. Specimens (including the cervical biopsies) that are not used for research testing immediately are processed and stored for future testing. Plasma, PBMCs, vaginal swabs, anal swabs, and CVL supernatants are stored at -80 °C. Any remaining specimens are stored for potential future use only if the patient has consented to storage of specimens (a check box is included on the informed consent document). These specimens may be used in the future to further evaluate HIV, immunologic and/or microbiologic factors of interest, or as a source of DNA for future genetic analysis.

Study data are recorded on case report forms (CRFs) and entered into a HIPAA compliant web-based password-protected relational database (REDCap). Additional study data are housed in a HIPPA-compliant web-based password-protected One Drive database (Microsoft 365 Educational Licensing, Redmond, WA). All study paper forms and materials are maintained in locked cabinets and all data files are password protected in accordance with Emory IRB. Variables are continuously examined for data entry errors, inconsistencies, incompleteness, and plausibility of outliers with any changes documented.

### Data analysis

Univariate analyses will evaluate for differences in pre-contraception participant characteristics by HC method (DMPA, ENG-implant, LNG-IUD). If meaningful differences are observed, inverse propensity score weighting will be used to weight participants so that the study arms are more similar on pre-contraceptive participant characteristics. To account for repeated measures with a participant, all analyses will use mixed models with an appropriate distribution (normal, beta) and link function (identity, log), use an unstructured covariance matrix, and include a random effect for participant. For Aim 1, the primary outcome of interest is proportion of CCR5 expressing CD4^+^ T cells, while secondary outcomes are other immunologic outcomes from the flow cytometry and immune-related protein assays characteristics that may provide deeper insight into immune regulation in the FGT. Separate models for each outcome will contain time (categorical: visit 1, 2, 3, 4, 5, 6, 7, 8), contraceptive method (DMPA, ENG-implant, LNG-IUD), and a method by time interaction term. Models will additionally include important covariates such as age, race, STI presence, menstrual cycle characteristics and, if needed, incorporate propensity score weights. Model-based estimates of the change in mean outcome level within a contraceptive method over time (visit 1 and 2 versus each of 3, 6, and 12 months after contraceptive administration) will be computed. If the interaction term of method by time is significant, model-based comparisons of the means by contraceptive method and time (visit 1 and 2 versus each of: 3, 6, and 12 months after contraceptive administration) will also be computed pairwise for all possible combinations. Statistical significance will be set at α = 0.05 for the primary outcome of CCR5 and the main effects (contraceptive method, time) and the interaction term (method by time). Secondary outcomes will be evaluated for direction and magnitude of the associations. Models will be stratified by each specimen type (blood, CVL, cytobrush, biopsy).

In a subset of individuals, for a total of 75 women (25 per arm), cytobrush and blood specimens at visit 1, 2, 5 and 6 will be stained for more markers using BD symphony. In addition to T cells, we are also conducting innate cell marker analysis. These data include total, activated (express CD80 and CD86) and exhausted (expression of PD-L1) B cells, dendritic cells (myeloid DCs and plasmacytoid DCs), monocytes (classical, intermediate, and non-classical), macrophages, neutrophils and myeloid-derived suppressor cells (MDSCs including monocytic MDSCs and neutrophilic MDSCs). Principal component analysis will be applied in an exploratory analysis to characterize patterns of specific cell responses that may better describe activation, proliferation, differentiation, and maturation of immune cells in the FGT following HC use. Computational tools help us visualize relationships in high dimensional space and separate rare cells into populations based on distinct, multi-feature phenotypes. This methodology compares analysis of the human cell surface immunophenotyping dataset by three methods: Method 1: Bivariate gating analysis employs pairwise comparisons of markers to select, or gate, cell populations. Method 2: Visualization of t-Stochastic Neighbor Embedding (viSNE) analysis. This is a computational tool that projects cells onto a two-dimensional map such that the distances between cells in 2D reflect the distance between them in high-dimensional space. Method 3: Spanning-Tree Progression Analysis of Density-Normalized Events (SPADE) analysis. With high content single-cell experiments measuring > 30 features per cell, sequential bivariate gating can overlook populations of cells with low or unexpected patterns of markers expression. SPADE clusters cells based on phenotypic similarities in high-dimensional space and creates a minimum-spanning tree that illustrates the relationships between cell populations in two dimensions.

For Aim 2, we will evaluate alpha diversity at each time point, using the Shannon diversity index and Chao1 measures. We will also investigate microbiome CST over time. We will use additional statistical tools, such as a Principal Component Analysis to visualize clustering of the data based on distance metrics (weighted Unifrac, Bray–Curtis) [75] and advanced tools such as scree plots to identify the specific taxa that account for group differences. We will evaluate for effect modification on the microbial outcomes, such as BV status (BV presence or absence based on Nugent Score), species diversity, CST, and specific bacterial species by including interaction terms, separately and stratified by contraceptive method. An indicator for each outcome and an outcome by time interaction term will be added to the contraceptive method stratified mixed models. If the outcome by time interaction term is significant, model-based comparisons of the means by outcome and time (visit 1 and 2 versus each of: 3, 6, and 12 months after contraceptive administration) will also be computed pairwise for all possible combinations. As the primary independent variable of interest is BV, statistical significance will be set at α = 0.05 for the main effects (BV, time) and interaction term (BV by time) from the repeated measures analysis for CCR5 expression.

Analyses will be conducted using SAS software (SAS Institute, Cary, NC) and R (R Core Team).

### Power/sample size

For Aim 1, our pilot longitudinal study among women treated with DMPA, LNG-IUD, and ENG-implant provided estimates utilized in sample size calculation. The pilot study evaluated CVL CCR5 levels at four different time points (two visits pre-contraceptive, two visits post-contraceptive) with *n* = 148 total visits [76]. At the pre-contraceptive time point, 23.0% (SD 16.1) of CVL CD4^+^ T-cells expressed CCR5. Post-contraceptive, mean percent of CD4^+^ T-cells expressing CCR5 was 38.3, 34.1 and 23.6 for DMPA, ENG-implant and LNG-IUD, respectively, which represents a 66%, 48% and 3% increase above the pre-contraceptive measurement, respectively [77]. Given 75 women in each contraceptive method arm (DMPA, LNG-IUD, ENG-implant) and allowing for 20% attrition produces a final sample size of 60 women per arm. Assuming the smallest clinically- meaningful change in the percentage of CD4^+^ T cells expressing CCR5 level is 50% and that change will only be observed for DMPA and ENG-implant (not LNG-IUD), produces an effect size of 0.3 (effect SD 2.7). This design achieves 92% power to test the HC method (DMPA, ENG-implant, LNG-IUD) by time (pre-/post-contraceptive) interaction using a Geisser-Greenhouse Corrected F test when significance level is 0.05. Secondary outcomes will be evaluated for direction and magnitude of the associations.

For Aim 2, using the same pilot data, 44% of visits (*n* = 76) had BV present [76]. BV status was consistent with the pre and in post time points (92% concordance) [76]. Mean percent of CD4^+^ T cells expressing CCR5 was 20.3 and 25.1, for no BV and BV present, respectively, with a standard deviation of 15.9 pre-HC and 29.8 and 37.0 with no BV and with BV, respectively, with a standard deviation of 22.1 post-HC [76]. With 75 women in each contraceptive method arm (DMPA, LNG-IUD, ENG-implant), and allowing for 20% attrition, we will have a final sample size of 60 women per arm. Assuming a 0.502 correlation between pre- and post-contraception measurements, 50% of time periods will have BV present, the smallest clinically-meaningful change in proportion of CD4 + T cells expressing CCR5 regardless of BV status is 50% and that a larger change will be observed for BV visits compared to non-BV visits (assuming 85% change vs. 15% change, respectively), producing an effect size of 0.46. This design achieves 87% power to detect a significant BV interaction for the association between each HC method used and CCR5 expression on CD4^+^ T cells using a Geisser-Greenhouse Corrected F test when significance level is 0.05.

## Discussion

The CHIME study design constitutes one of the most comprehensive prospective studies in the field, characterizing immune changes among 225 HIV-uninfected women longitudinally with complementary FGT sampling approaches [15, 77, 78]. Although a randomized controlled study design ensures balance between arms and is therefore commonly adopted in studies of this nature, for feasibility in the US, where a broad contraceptive mix is available and because contraceptive choice is a personal decision, the specific contraceptive type in this study will be left to the preference of participants. However, limitations from non-randomization are minimized by: [1] collecting and controlling for covariates that may impact immune response, including demographics, body mass index (BMI), current STIs, underlying health conditions, vaginal practices (e.g., douching), medication exposure, and semen exposure that could influence outcomes of interest; [2] evaluating two timepoints for each participant prior to contraceptive initiation intended to correspond to follicular and luteal phases, as the optimal non-contraceptive control for comparison; and [3] propensity score methods to adjust for pre-contraceptive characteristics that may be unevenly distributed by contraceptive arm such as STIs as well as behavioral and demographic characteristics.

By design, the study does not directly address the risk of HIV acquisition from hormonal contraceptives; rather, it explores surrogates that might provide a mechanistic explanation for this purported impact of HC use. Being exploratory in nature, the sample size is small, and we may not be able to detect small differences in some of the outcomes. We have carefully selected our markers to focus on our study aims; however, we acknowledge that additional markers may be of interest and can be explored in future evaluations. Furthermore, we acknowledge that many other factors that influence HIV susceptibility warrant further investigation, such as epithelial integrity and endogenous microbicides. We plan to use these banked samples to explore other potential mechanisms in future research. The data generated from the successful completion of this study will be instrumental in the design of a future prospective contraceptive trial to explore HIV acquisition risk and establish safe family planning options for high-risk women.

The COVID-19 pandemic presented unforeseeable challenges for clinical research. Beginning in March 2020, there was a temporary suspension of all research followed by a phased re-initiation of approved studies under stringent social distancing guidelines. During this time, healthcare providers and researchers were required to shift priorities to address and adapt to the immediate needs of the pandemic, including supplementing the existing workforce and staffing COVID wards. These and other personal pressures, such as childcare responsibilities, resulted in staff turnover that coincided with an administrative hiring freeze. While onboarding new staff, the study team focused on maintaining study visits for enrolled participants and processing samples. At the same time, many of our enrolled participants relocated due to job loss, school closures or other COVID-related factors. Those that did not relocate expressed hesitancy regarding attending in-person study visits. Collectively, the pressures of the pandemic on participants resulted in early study terminations or missing visits during the pandemic, requiring the investigative team to re-examine the planned statistical approach and adopt methods that were more robust to missing data. Despite these challenges and limitations, the rich phenotypic and immunologic data collected as part of the CHIME study is poised to help us understand the role of endogenous and exogenous ovarian hormones in regulating immunologic function in the female genital tract.

## Data Availability

The data from study are available from the corresponding author upon reasonable request.

## Abbreviations

BV: Bacterial vaginosis
CPT: Cell preparation tubes
CRF: Case report forms
CST: Community state type
CVL: Cervicovaginal lavage
DMPA: Depot medroxyprogesterone acetate
ECHO trial: Evidence for Contraceptive Options and HIV Outcomes trial
ENG: Etonogestrel
FACS: Fluorescence-activated cell sorting
FGT: Female genital tract
FSH: Follicle-stimulating hormone
HC: Hormonal contraception
HIV: Human immunodeficiency virus
HPO: Hypothalamic-pituitary-ovarian axis
HSV: Herpes simplex virus
IUD: Intrauterine device
LEEP: Loop electrosurgical excision procedure
LH: Luteinizing hormone
LNG: Levonorgestrel
LLOD: Lower limit of detection
MPA: Medroxyprogesterone acetate
NAAT: Nucleic acid amplification test
PBMC: Peripheral blood mononuclear cells
PBS: Phosphate-buffered saline
PSA: Prostate-specific antigen
RPM: Rotations per minute
RPR: Rapid plasma reagin
RT: Room temperature
STIs: Sexually transmitted infections
